# Hyperhomocysteinemia is associated with the risk of venous thromboembolism in patients with mental illness: a case-control study

**DOI:** 10.3389/fpsyt.2024.1340138

**Published:** 2024-05-17

**Authors:** Jiaoyan Wang, Yingchun Zhang, Keming Ren, Yeping Li, Kejing Ying

**Affiliations:** ^1^ Division of Pulmonary and Critical Care Medicine, Wenzhou Medical University Affiliated Taizhou Hospital, Linhai, Zhejiang, China; ^2^ Department of Pulmonary and Critical Care Medicine, Regional Medical Center for National Institute of Respiratory Diseases, Sir Run Run Shaw Hospital, School of Medicine, Zhejiang University, Hangzhou, China; ^3^ Department of mental health, Sir Run Run Shaw Hospital, Zhejiang University School of Medicine, Hangzhou, China

**Keywords:** mental illness, venous thromboembolism, hyperhomocysteinemia, hyperprolactinemia, pulmonary thromboembolism

## Abstract

**Objective:**

The risk of venous thromboembolism in patients with mental illness has been insufficiently addressed. This study aimed to assess the correlation between hyperhomocysteinemia and venous thromboembolism prevalence among this population.

**Methods:**

Patients with a diagnosis of mental illness and concurrent venous thromboembolism, admitted to Sir Run Run Shaw Hospital at Zhejiang University School of Medicine between January 2014 and December 2021, were included in the venous thromboembolism group. The control group, approximately twice the size, comprised individuals with mental illness but without venous thromboembolism. Basic clinical data were gathered for both cohorts.

**Results:**

In psychiatric patients, elevated D-dimer levels(OR=5.60,95% CI 3.28–10.00), hyperhomocysteinemia (OR=2.37,95% CI 1.10–5.14), and hyperprolactinemia(OR= 2.68,95% CI 1.12–6.42)were significant risk factors for venous thromboembolism. According to further subgroup analyses, hyperhomocysteinemia is a significant risk factor associated with pulmonary embolism, with an OR of 5.08 (95% CI 1.20–21.48). An interaction effect between gender and homocysteine level was found, with a p-interaction of 0.022. A subsequent analysis confirmed the association between hyperhomocysteinemia and venous thromboembolism in female psychiatric patients, with an OR of 3.34 (95% CI 1.68–6.65), indicating that hyperhomocysteinemia is a significant risk factor for venous thromboembolism in women.

**Conclusion:**

Patients with psychiatric disorders were found to have an elevated risk of venous thromboembolism, which was associated with increased levels of D-dimer, hyperprolactinemia, and hyperhomocysteinemia. A strong correlation between hyperhomocysteinemia and pulmonary embolism was identified in patients with mental illnesses. Furthermore, the study revealed that female psychiatric patients with hyperhomocysteinemia constituted a high-risk group for venous thromboembolism. This finding holds significant clinical implications, suggesting that early preventative measures could be implemented for this high-risk population to reduce the incidence of thromboembolic events during hospitalization for psychiatric patients.

## Introduction

Venous thromboembolism (VTE) is a common thromboembolic disease in the population, mainly including Deep Venous Thrombosis (DVT) and Pulmonary Thromboembolism (PTE) (1). The incidence of VTE is high all over the world, some studies had found that the annual incidence of VTE could be as high as 2 ‰ (1), A study found that the hospitalization rate of venous thromboembolism in China increased about five times between 2007 and 2016, and the mortality rate of VTE was seen to rise with age over time (2). Due to the high morbidity and mortality of VTE, it has become a public social health problem.

Patients with mental illness often suffer from social withdrawal and reduced physical activity due to their illness, and most patients with obvious psychiatric symptoms need to take antipsychotic drugs to control clinical symptoms, all of which can lead to blood hypercoagulation and promote the formation of venous thrombosis (3, 4). Previous research (5, 6) has found that the overall prevalence of venous thrombosis in hospitalized patients with mental illness was as high as 1–2%. However, concerning the incidence of venous thromboembolism (VTE) in individuals with mental disorders, the current body of research is scant, and no consensus on incidence rates has been established. Consequently, to ascertain the actual incidence rates of VTE among this patient population, future studies necessitate a more comprehensive and profound investigation. Nevertheless, the fact that mental patients are easy to be complicated with VTE has not attracted enough attention in clinics, which often leads to missed diagnosis and adverse clinical outcomes.

Homocysteine is an amino acid that relies on the vitamin B6 and vitamin B12 pathways for synthesis during methionine metabolism. Homocysteine can cause oxidative stress and promote vascular inflammation through the activation of various signaling pathways, which in turn causes vascular damage (7) and induces thrombus formation. It has been found that the increase of plasma homocysteine concentration is a risk factor for many cardiovascular diseases, which significantly increases the risk of atherosclerosis and contributes to the formation of arterial thrombosis (7–9). Additional research revealed that hyperhomocysteinemia is a stand-alone risk factor for VTE (10), However, studies on the correlation between homocysteine and VTE were mostly retrospective analyses (11, 12).

Several studies have found that the concentration of serum homocysteine in patients with mental disorders such as schizophrenia, depression and Alzheimer’s disease was higher than that in the general population (13–15). Additionally, hyperhomocysteinemia may contribute to the onset and progression of certain mental illnesses.

At present, the attention caused by mental illness patients with VTE is still low, and there are few studies on the risk factors of mental illness patients with VTE. Referring to the previous literature, it is found that the incidence of HHcy in mental illness patients is much higher than that of the general population. It is speculated that HHcy may be a related risk factor for mental illness patients with VTE. Therefore, this paper intends to study the correlation between HHcy and VTE in patients with mental illness.

## Material and methods

### Study population

**Figure 1:**
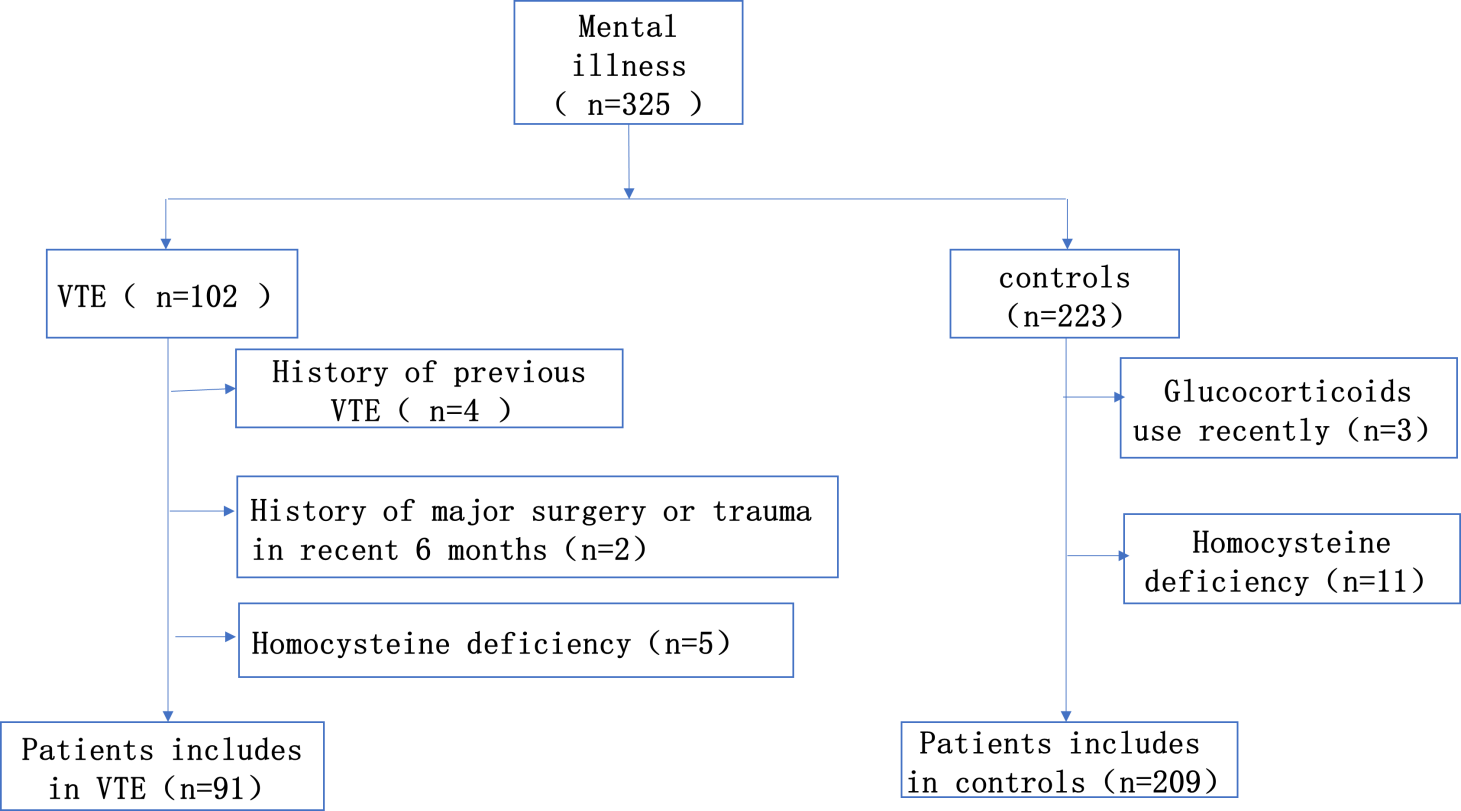
Flowchart of the study population.

This is a single-center retrospective case-control study. From January 2014 to December 2021, we continuously enrolled 91 consecutive patients diagnosed with mental illness combined with VTE in Sir Run Run Shaw Hospital, Zhejiang University School of Medicine. Inclusion criteria for the case group: Patients with a diagnosis of both mental illness and VTE, with a diagnosis of mental illness that meets the diagnostic criteria of the International Classification of Diseases, 10th edition (ICD-10) or the American Manual of Mental Disorders and Statistics, 5th edition (DSM-V) (16). The diagnosis of deep vein thrombosis was dependent on Doppler ultrasound. And the diagnosis of pulmonary embolism was confirmed by CT pulmonary angiography. Any patients who fit one of the following descriptions were disqualified: 1. Patients diagnosed with venous thromboembolism prior to admission; 2. Patients who had taken contraceptives, folic acid, vitamin B12 and glucocorticoids within the last 6 months; 3. Patients were recently pregnant or breastfeeding; 4. Patient had a history of major surgery or trauma within the last 6 months. The appropriate subjects were selected from the psychiatric patients without VTE in the same period as the control group, the proportion was about 1:2. The study was approved by the Ethics Committee (**Figure 1**).

### Clinical data collection and definitions

The patient’s sex, age, body mass index (BMI), smoking history, antipsychotic use, olanzapine use, antidepressant use, Complications (hypertension, diabetes, chronic kidney disease, malignant tumor, coronary heart disease, cerebral infarction) were obtained and collected from the hospital medical record system. Routine blood tests and blood biochemistry were performed upon admission to obtain the following laboratory results such as neutrophils, lymphocytes, fibrinogen, platelets, D-dimer, CRP, homocysteine, prolactin and so on. Hyperhomocysteinemia is defined as plasma homocysteine levels >15 umol/L (17). Fasting blood samples were collected on admission. Hyperprolactinemia is defined as a single plasma prolactin level above the upper limit of normal (18), the upper limit of normal prolactin for men and postmenopausal women in our hospital was set at 20 μg/L.

### Statistical analysis

Statistical analysis was conducted using SPSS 26.0 and R 4.2.2 software. Categorical data were presented as frequencies and analyzed by chi-squared test or Fisher’s exact test as appropriate. Continuous data were expressed as mean ± SD or median with interquartile range according to the normality of the distributions. To test the differences between the groups, t-test was applied to continuous data that conform to a regular distribution, the Mann–Whitney U-test was used for continuous data that did not conform to a regular distribution. The concentration of plasma homocysteine was analyzed by categorical variables with 15 umol/L as the boundary. Logistic regressions were used to assess the relationship between homocysteine and VTE. On the basis of univariate analysis followed by Multivariate logistic regression analysis (including independent variables with P values <0.1 and sex) to explore the independent risk factors for VTE in patients with mental illness. We then explored the effect of hyperhomocysteinemia on VTE according to the VTE subtype typing. It has been shown that there is a difference between elevated plasma homocysteine levels and the occurrence of VTE in different genders (19, 20). Then the interaction between gender and Hcy is analyzed by R4.2.2 software, the p value of the interaction is calculated, and the bar chart of the interaction between Hcy and gender is drawn by using the cat_plot function in R package to visualize, and then stratified analysis is carried out according to gender. Lastly, we computed the prediction probability of combined D-dimer, homocysteine and prolactin by logistic regression model, and plotted the predictive probability value and ROC curve of VTE to evaluate the predictive performance of combined three indicators in patients with mental illness complicated with VTE. In this study, the definition of P < 0.05 is statistically significant.

## Results

As shown in **Table 1**, A total of 91 patients were included in VTE group, 27 (29.7%) males and 64 (70.3%) females, with a mean age of 66 years. The control group included a total of 209 cases, 63 (30.1%) males and 146 (69.9) females, with a mean age of 61 (55–68) years. There were no significant differences in BMI, Antidepressant, Lymphocyte, Platelet, TG, LDL, HDL and Physical restraint between the 2 groups. However, age, smoking history, antipsychotic use, olanzapine use, DDI, hsCRP, neutrophils, fibrinogen, homocysteine and prolactin were found to be statistically significant in the cases and control groups (p < 0.05).

**Table 1:** Baseline characteristics of venous thrombosis cases and controls.

		VTE cases(n=91)	controls(n=209)	P value
Sex				
	male	27(29.7)	63(30.1)	0.934
	female	64(70.3)	146(69.9)	
Age(year)*		66(62–72)	61(55–68)	0.000
Smoking*				
	no smoking	81(89.0)	188(90.0)	0.045
	previous smoking	8(8.8)	7(3.3)	
	current smoking	2(2.2)	14(6.7)	
BMI(kg/m2)				
	< 18.5	6(6.6)	25(12.0)	0.326
	18.5- 24.9	64(70.3)	139(66.5)	
	25.0- 29.9	21(23.1)	41(19.6)	
	≥30	0	4(1.9)	
Antipsychotic*				
	none	29(31.9)	111(53.1)	0.001
	first generation	0	0	
	Second generation	62(68.1)	98(46.9)	
	both	0	0	
Olanzapine*		50(54.9)	72(34.4)	0.001
Mental illness				
	Depression	72(79.1)	164(78.5)	
	Non-depressive	19(20.9)	45(21.5)	0.899
Antidepressant		76(83.5)	170(81.3)	0.652
DDI(ug/mL) *		1.47(0.89–2.32)	0.29(0.23–0.44)	0.000
hsCRP(mg/L)*				
	≥6	12(13.2)	4(1.9)	0.000
Neutrophil (/L) *				
	<1.8*10^9	3(3.3)	15(7.2)	
	1.8–6.3*10^9	79(86.8)	189(90.4)	0.009
	>6.3*10^9	9(9.9)	5(2.4)	
Lymphocyte (/L)				
	<1.1*10^9	14(15.4)	26(12.4)	0.860
	1.1–3.2*10^9	76(83.5)	179(85.6)	
	>3.2*10^9	1(1.1)	4(1.9)	
Fibrinogen (g/L) *				
	<2	4(4.4)	8(3.8)	
	2–4	80(87.9)	198(94.7)	0.023
	>4	7(7.7)	3(1.4)	
Platelet (/L)				
	<125 *10^9	9(9.9)	10(4.8)	
	125–350*10^9	81(89.0)	197(94.3)	0.180
	>350 *10^9	1(1.1)	2(1.0)	
Homocysteine(umol/L)*				
	≤15	55(60.4)	158(75.6)	
	>15	37(40.7)	51(24.4)	0.004
Prolactin(μg/L)*				
	≤20	65(71.4)	175(83.7)	
	>20	27(29.7)	34(16.3)	0.008
TG(mmol/L)				
	>1.7	39(42.9)	67(32.1)	0.072
HDL(mmol/L)				
	<1.03	18(19.8)	49(23.4)	
	1.03–1.55	66(72.5)	131(62.7)	0.188
	>1.55	7(7.7)	29(13.9)	
LDL(mmol/L)				
	<1.89	3(3.3)	14(6.7)	
	1.89–4.21	84(92.3)	182(87.1)	0.394
	>4.21	4(4.4)	13(6.2)	
Physical restraint		3(3.3)	2(1.0)	0.166
Complication				
	Hypertension	45(49.5)	85(40.7)	0.158
	Diabetes	11(12.1)	26(12.4)	0.932
	CKD	2(2.2)	4(1.9)	1.000
	Cancer	6(6.6)	19(9.1)	0.472
	CHD*	15(16.5)	13(6.2)	0.005
	CI*	19(20.9)	15(7.2)	0.001

Continuous variables are shown as median (25th percentile– 75th percentile). Categorical variables are shown as percentages with numbers in brackets. P Value for continuous variables were obtained from Mann-Whitney U Test(age and DDI).P Value for categorical variables were obtained using χ2 test or Fisher’s Exact Test.

VTE, venous thromboembolism; BMI, body mass index; DDI, D-Dimer; hsCRP, high- sensitivity C- reactive protein; TG, Triglyceride; HDL, high-density lipoprotein; LDL, low-density lipoprotein; CKD, Chronic kidney disease; CHD, Coronary heart disease; CI, Cerebral infarction.

*indicates a statistical difference between the two groups, P value<0.05.

**Table 2** showed the multifactorial regression analysis of the occurrence of VTE in patients with mental illness. With the occurrence of VTE event as the dependent variable, independent variables with P value<0.1 in the univariate analysis (age, smoking history, antipsychotic use, olanzapine use, D-dimer, CRP, neutrophils, fibrinogen, homocysteine, prolactin, triglycerides, coronary artery disease, cerebral infarction) and sex were included in the multifactorial regression model. The results showed that DDI (OR=5.60, 95%CI3.28–10.00), hyperhomocysteinemia (OR=2.37, 95%CI1.10–5.14) and hyperprolactinemia were statistically different between the cases and controls groups.

**Table 2:** Logistic regression analysis for risk factors attributing to VTE presence in the study.

		OR(95%CI)	P value
Sex		0.56(0.22–1.38)	0.206
Age(year)		1.05(0.999–1.09)	0.057
Smoking			
	No smoking	Reference	0.301
	Previous smoking	2.40(0.44–13.07)	
	Current smoking	0.35(0.05–2.67)	
Antipsychotic		1.58(0.90–2.77)	0.114
Olanzapine		1.31(0.44–3.84)	0.627
DDI(ug/mL) *		5.60(3.28–10.00)	0.000
hsCRP(mg/L)		3.43(0.61–19.23)	0.161
Neutrophil (/L)			
	<1.8*10^9	0.75(0.15–3.81)	
	1.8–6.3*10^9	Reference	0.788
	>6.3*10^9	1.68(0.29–9.73)	
Fibrinogen (g/L)			
	<2	0.92(0.15–5.62)	
	2–4	Reference	0.849
	>4	1.97(0.19–20.66)	
Homocysteine(umol/L)*		2.37(1.10–5.14)	0.028
Prolactin(μg/L)*		2.68(1.12–6.42)	0.027
TG(mmol/L)		1.51(0.71–3.20)	0.276
CHD		1.78(0.50–6.28)	0.371
CI		1.97(0.71–5.50)	0.195

DDI, D-Dimer; hsCRP, high- sensitivity C- reactive protein; TG, Triglyceride; CHD, Coronary heart disease; CI, Cerebral infarction.

*indicates a statistical difference between the two groups, P value<0.05.

In multivariable-adjusted regression models, homocysteine was the biomarker that was positively related with incident VTE, we further analyzed the association between hyperhomocysteinemia and the various subtypes of VTE (DVT group, PE group, DVT+PE group) (**Table 3**). In the unadjusted model, hyperhomocysteinemia was associated with DVT (OR 1.86, 95% CI 1.02– 3.38) and PE (OR 2.82, 95% CI 1.13–7.02). Both Model 1 (adjusted for age, prolactin and DDI) and Model 2 (adjusted for sex, age, TG, hsCRP, antipsychotic, prolactin and DDI.)showed a trend of increasing ORs among groups with DVT, PE, and DVT+PE. Model 2 showed a strong correlation between hyperhomocysteinemia and PE, with an OR of 5.08 (95%CI,1.20–21.48). In the DVT+PE group, the odds ratio (OR) was 39.31 with a 95% confidence interval (CI) of 0.63–2437, which was statistically indistinguishable. This indistinction may be attributed to the subgroup’s limited sample size. Consequently, the findings do not conclusively negate the involvement of hyperhomocysteinemia in DVT+PE. Future studies will aim to substantiate this correlation by enlarging the DVT+PE group’s sample size.

**Figure 2:**
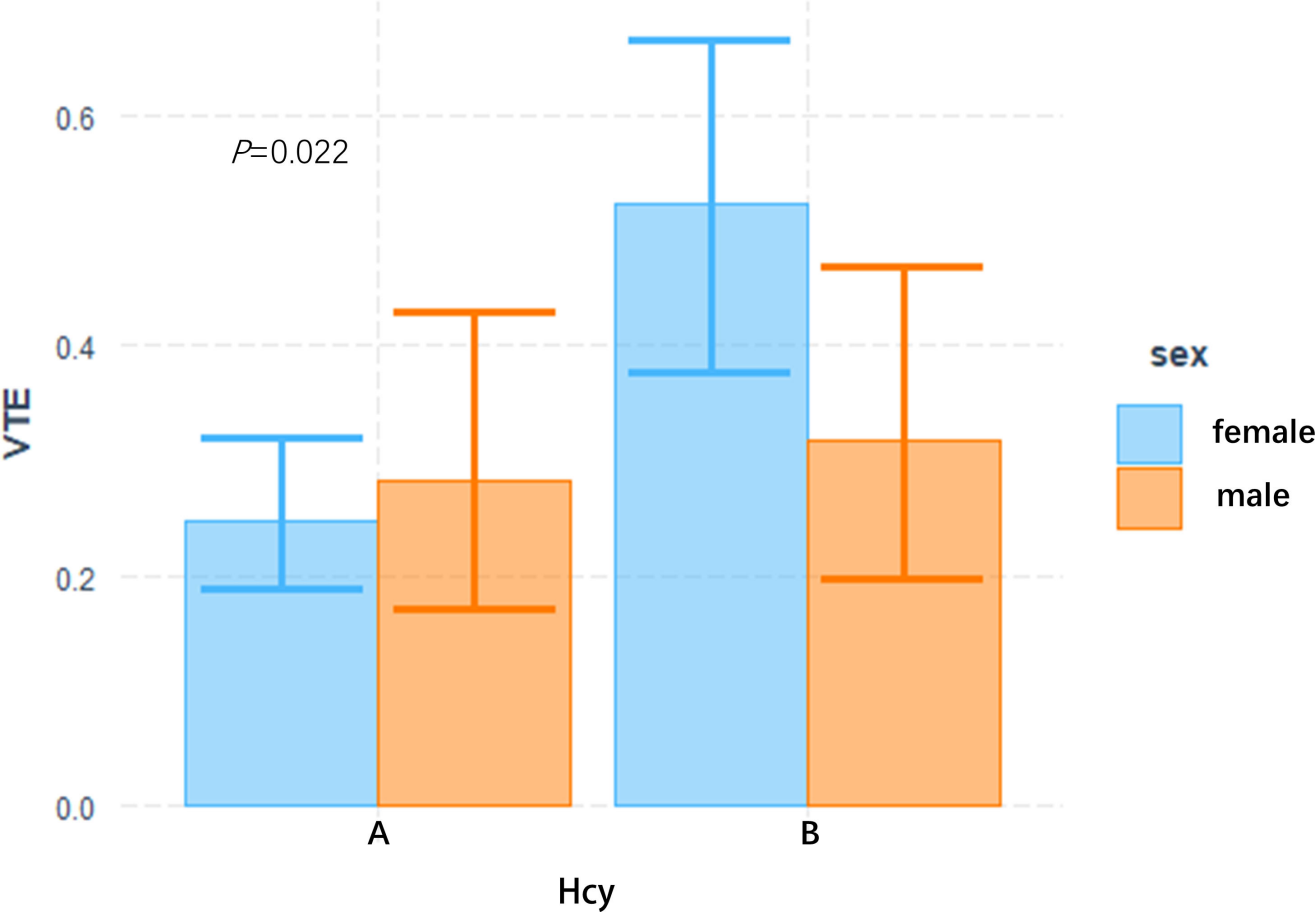
Interactive analysis of HCY and gender bar chart group A: plasma Hcy ≤ 15 umol/L, B group: plasma Hcy > 15 umol/L.

**Figure 3:**
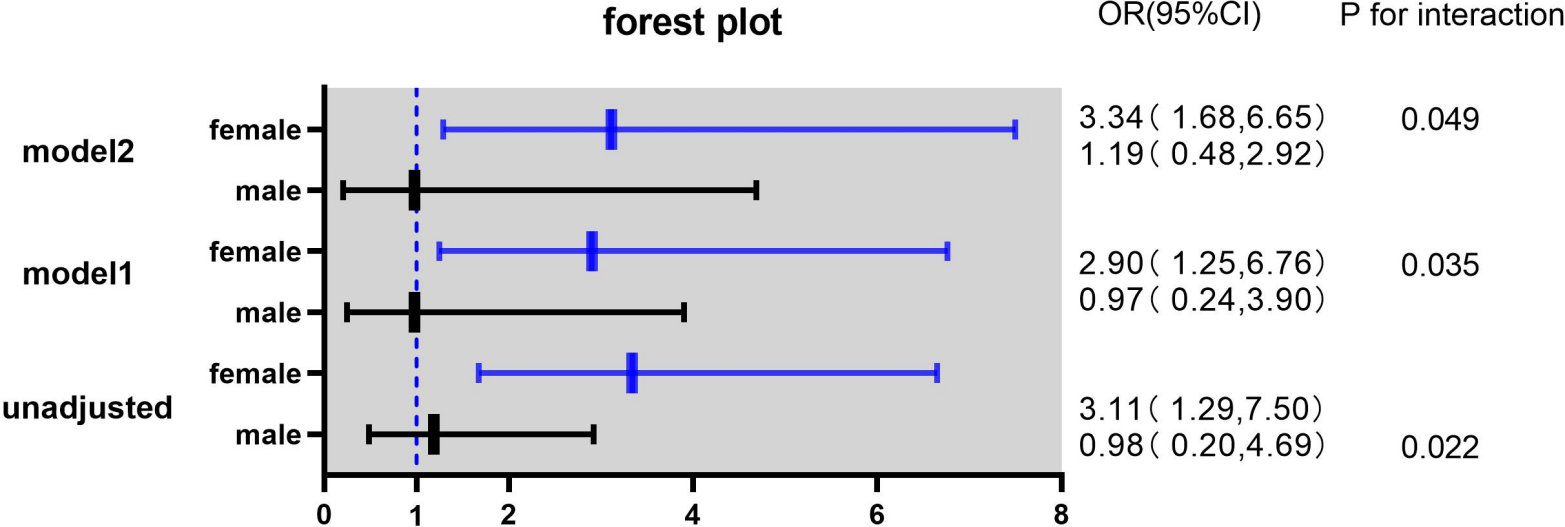
Odds ratios (ORs) with 95% confidence intervals (CIs) for overall venous thromboembolism according to sex.

In this study, the interaction between homocysteine and sex was further analyzed, the p value of the interaction was calculated by R4.2.2 software, and the bar chart of the interaction between homocysteine and sex was drawn by using the cat_plot function in R packet, showing the interaction effects between homocysteine and sex. The y-axis denotes the VTE incidence rates. At plasma homocysteine concentrations ≤15 µmol/L, both male (0.28) and female (0.25) patients exhibited comparable rates of venous thromboembolism. Contrastingly, at levels >15 µmol/L, a pronounced divergence in incidence rates was observed between genders. Notably, the rate for female patients escalated to 0.52, a stark increase, whereas the rate for male patients remained constant at 0.31, similar to prior data. Statistical analysis revealed the p-interaction was 0.022, thereby confirming a statistically significant interaction between gender and homocysteine levels. Considering the significant interaction observed between gender and homocysteine levels, a subgroup analysis was performed. It was found that hyperhomocysteinemia was significantly correlated with VTE in female patients with mental disorders, which was a risk factor for VTE in female patients with mental disorders, and after adjusting multiple confounding factors (age, prolactin, D-dimer, triglyceride, CRP, antipsychotics). It still has a strong correlation, OR=3.34 (95%CI=1.68–6.65). On the contrary, hyperhomocysteinemia was not associated with the occurrence of VTE in male patients with mental illness, OR=1.19 (95%CI=0.48–2.92). **Figures 2**, **3**

Model 1 adjusted for age, prolactin and DDI.

Model 2 adjusted for age, TG, hsCRP, antipsychotic prolactin and DDI.

**Figure 4:**
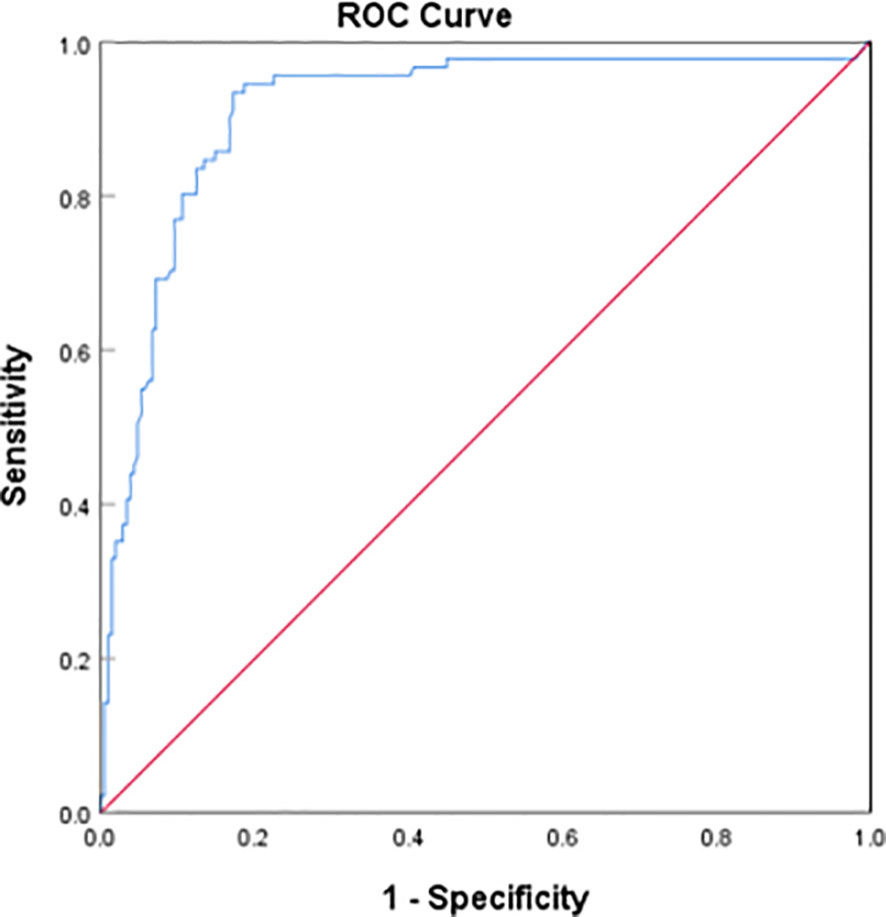
Receiver operating characteristic (ROC) plot of DDI+hyperhomocysteinemia + hyperprolactinemia predicting presence of VTE AUC area was 0.912 (95%CI:0.873–0.951).

Through the binary Logistic regression model in SPSS26.0, the prediction probability of VTE was calculated by combining the three laboratory indexes of D-dimer, homocysteine and prolactin, and then the ROC curve of prediction probability and VTE was drawn (**Figure 4**). The AUC area was 0.912 (95%CI:0.873–0.951), and the sensitivity and specificity of the best cutoff value was 0.934 and 0.828 respectively. It is suggested that the combination of D-dimer, homocysteine and prolactin has high diagnostic value for VTE in patients with mental disorders.

**Table 3:** Associations Between hyperhomocysteinemia and VTE(DVT、PE、DVT+PE).

VTE	cases	controls	Unadjusted	Model 1	Model 2
			OR(95%CI)	OR(95%CI)	OR(95%CI)
DVT(n=64)					
Hyperhomocysteinemia	24(37.5)	51(24.4)	1.86(1.02–3.38)	1.97(0.93–4.15)	2.09(0.96–4.56)
PE(n=21)					
Hyperhomocysteinemia	10(47.6)	51(24.4)	2.82(1.13–7.02)	3.68(0.99–13.69)	5.08(1.20–21.48)
DVT+PE(n=6)					
Hyperhomocysteinemia	3(50.0)	51(24.4)	3.01(0.61–15.83)	13.19(1.28–135.83)	39.31 (0.63–2437.51)

VTE, venous thromboembolism; DVT, deep vein thrombosis; PE, pulmonary embolism.

Model 1 adjusted for sex, age, prolactin and DDI.

Model 2 adjusted for sex, age, TG, hsCRP, antipsychotic, prolactin and DDI.

## Discussion

In this study, it was found that hyperhomocysteinemia, hyperprolactinemia and elevated D-dimer were the risk factors of VTE in patients with mental illness, and after adjusting for a variety of confounding factors, the above three indexes were still strongly associated with the occurrence of VTE in patients with mental illness. Subsequently, the correlation of hyperhomocysteinemia among VTE subgroups was further studied, and it was found that hyperhomocysteinemia was a risk factor for DVT and PE in patients with mental illness in the model with unadjusted confounding factors. After adjusting for confounding factors, the correlation of hyperhomocysteinemia increased in DVT group, PE group and DVT+PE group, but only the p value of PE group was < 0.05. However, due to the relatively small number of cases in the subgroup analysis, there may be some bias, which leads to the underestimation of the relationship between HHcy and DVT+PE group and DVT group, so a larger sample is needed in the future. In addition, this study also found that there was an interaction between homocysteine and gender. Hyperhomocysteinemia significantly increased the risk of VTE in female patients with mental illness, and after adjusting for confounding factors, the interaction still existed. The follow-up stratified analysis gives more accurate results, suggesting that female mental patients with hyperhomocysteinemia are at high risk for VTE. The three factors of hyperhomocysteinemia, hyperprolactinemia and D-dimer were included in the ROC curve, and the AUC area was 0.912, suggesting that it has important reference value for the diagnosis of VTE in patients with mental disorders.

In recent years, more and more researchers were concerned that patients with mental illness were prone to co-occurring VTE with atypical clinical symptoms, which were easily missed. The reduction of activity caused by mental illness itself and the use of antipsychotic drugs during treatment can greatly increase the risk of venous thrombosis in patients with mental illness (21–23). However, in terms of clinical diagnosis and treatment, the attention to mental illness complicated with VTE is still low, and there is a high rate of missed diagnosis, which can easily lead to poor prognosis of patients.

However, the risk factors for the occurrence of venous thrombosis in patients with mental illness remain unclear and may be associated with the mental illness itself and the administration of antipsychotic drugs.

Homocysteine is an important enzyme in the regulation of methionine and cysteine metabolism. On the one hand, homocysteine levels are strongly correlated with psychiatric symptoms and patient prognosis, and they play a crucial role in the development of mental illness, particularly schizophrenia (24, 25). On the other hand, hyperhomocysteinemia is a risk factor for cardiovascular diseases, which can induce endothelial cell injury and apoptosis by inhibiting the synthesis and repair of DNA through multiple pathways (26). It can also interfere with NO metabolism of endothelial cells, lead to the imbalance of NO metabolism (27) and induce endothelial cell dysfunction. At the same time, homocysteine can directly or indirectly activate platelets, promote platelet adhesion and aggregation with a variety of blood cells and endothelial cells, and regulate the contraction of blood clots (28). In general, hyperhomocysteinemia may affect coagulation and fibrinolysis in the body from multiple pathways.

A review of previous studies shows that most studies believe that hyperhomocysteinemia is an independent risk factor for VTE and increases the risk of venous thrombosis. Multiple meta-analyses (11, 29) suggested that hyperhomocysteinemia increased the incidence of venous thrombosis in patients. The latest meta-analysis found that the increase of plasma total homocysteine level was closely related to venous thrombosis. The incidence of VTE increased by 60% for every 5 mol/L increase in plasma homocysteine(OR=1.6, 95% CI, 1.10–2.34), which might be associated with the methylenetetrahydrofolate reductase gene 677TT (MTHFR 677TT) mutation (11). MTHFR is a key enzyme in homocysteine metabolism, When its gene mutation occurs, it will lead to the weakening of related enzyme activity and the continuous accumulation of homocysteine in the blood, resulting in hyperhomocysteinemia (30). However, the above studies do not adjust the influence of common confounding factors such as age, sex, smoking history and BMI, so the conclusions may be biased. A cohort study (31) found that elevated plasma homocysteine levels were significantly associated with the future occurrence of VTE. Patients with homocysteine level ≥ 12.9 μ mol/L had a 0.3-fold increased risk compared with patients with homocysteine level ≤ 8.6 μ mol/L, with a HR of 1.31 (95%CI:1.06–1.63). Further classification of PE and DVT revealed that elevated homocysteine levels were associated with unprovoked PE (HR=2.13 (95% CI, 1.30–3.51) and unprovoked DVT (HR=1.59 (95% CI, 1.05–2.40).

However, some studies have come to the opposite conclusion. A case-control study in the Netherlands included 1,689 cases with two separate control groups, one for general population controls and the other for partner-matched controls. The case group was compared with the two groups respectively. After adjusting the common confounding factors by logistic regression, the level of homocysteine had nothing to do with VTE. Further stratification was carried out according to sex, DVT, PE, inductive VTE and non-inductive VTE. It was still found that the increase of homocysteine concentration did not increase the incidence of thrombosis in patients (32).

There is a paucity of related research in patients with mental illness complicated with VTE. A variety of mental illness can cause an increase in the concentration of plasma homocysteine in patients (13, 14). An observational study focusing on psychiatric disorders with VTE found that the most common laboratory risk factors for patients were elevated FVIII levels and hyperhomocysteinemia, with approximately 33% of patients having hyperhomocysteinemia (33). A meta-analysis (34) shows that about 30% of patients with depression have HHcy, accompanied by a decrease in folic acid levels, which is much higher than the proportion of hyperhomocysteinemia in the general population. A case-control (35) study included 93 patients with schizophrenia and 60 healthy volunteers found that patients with schizophrenia had lower concentrations of tetrahydrobiopterin and folic acid and higher levels of Hcy than healthy volunteers. Some studies have found that the prevalence of HHcy in patients with schizophrenia can reach 30–50% (13, 36). A population screening program in China, which included 110551 subjects from 31 provinces across the country, found that only about 8 per cent of patients had HHcy (37). In conclusion, the prevalence of HHcy in patients with mental illness is substantially higher than that in the general population.

Therefore, this study explored the relationship between hyperhomocysteinemia and VTE in patients with mental illness. In this study, the proportion of hyperhomocysteinemia in the case group and the control group was 40% and 24% respectively, which was much higher than that in the normal population (8%). This is consistent with the conclusion that the incidence of hyperhomocysteinemia in patients with mental illness is higher than that in the general population.

It was also found that hyperhomocysteinemia was indeed a risk factor for VTE in patients with mental illness, and it still had a high risk ratio after adjusting confounding factors. Further analysis found that there was a strong correlation between hyperhomocysteinemia and the occurrence of PE in patients with mental illness. To investigate the correlation between homocysteine levels and DVT + PE group, a larger sample study is needed in the future.

In addition, there may be gender differences in the risk of VTE in hyperhomocysteinemia. The HUNT2 study from Norwegian (38) found that the risk of VTE was approximately doubled in male patients with plasma homocysteine concentrations above the 95th percentile, OR= 2.17 (95% CI 1.20–3.91), whereas no association was observed in female patients, OR=1.00. However, the stratified analysis of Tsai et al. (12) found that hyperhomocysteinemia was strongly correlated with the incidence of VTE in younger patients (<65 years old), female, Caucasian, and non-diabetic patients.

In this paper, we examined the interaction between homocysteine levels and gender, which showed that an interaction was present between homocysteine levels and gender with a p-interaction value of less than 0.05. This confirmed the statistical significance of the gender-based interaction analysis outlined in the article. Further, subgroup analyses substantiated these findings. The study indicated that female patients suffering from mental disorders with elevated levels of homocysteine faced a significantly high risk of developing venous thromboembolism (VTE). The OR value calculated in the model after adjusting for confounding factors was 3.34 (95%CI=1.68–6.65). There were some differences between this study and previous studies, the reasons may be as follows: 1. In the gender stratification analysis of hyperhomocysteinemia, the Norwegian study only explored the situation where the level of homocysteine was higher than 95 percentile (above 25.9 μ mol/l), which was different from the 15 μmol/L set in this study. 2. Different studies were aimed at different races and people, most of the previous research subjects are Caucasians, while the subjects of this study were Asians, and all of them were patients with mental illness. 3. There were certain selection bias and mixed bias in different studies.

Although a number of studies have shown that hyperhomocysteinemia is associated with the occurrence of VTE, clinical trials have found that reducing homocysteine therapy can not reduce the incidence of VTE in patients. The VITRO study (39) aimed to examine the effects of vitamin B supplementation on the reduction of DVT and PE by reducing homocysteine. The study recruited 701 patients with confirmed VTE (including 360 patients with hyperhomocysteinemia and 341 patients with normal homocysteine). Patients were randomized to the vitamin treatment and placebo treatment groups, and after 2.5 years of treatment and follow-up, vitamin treatment was found to reduce homocysteine levels in the hyperhomocysteine and normal homocysteine groups by 46% and 33%, but did not reduce the risk of recurrent venous thrombosis in the patients.

However, there are no randomized clinical trials on VTE in patients with mental disorders, and it is impossible to determine the correlation between lowering homocysteine levels with B vitamins and VTE in patients with mental disorders.

In this study, we plotted the ROC curve by combining DDI with homocysteine and prolactin for the first time. The value of DDI in the prediction of VTE had been unequivocally confirmed. A review of the previous literature found that a variety of antipsychotic drugs can lead to increased prolactin levels in patients (40, 41). It was found that the level of serum prolactin increased significantly in patients with mental illness treated with antipsychotics, and the increase of prolactin was positively correlated with the levels of DDI and fibrin/fibrinogen degradation products, which would increase the concentration of coagulation markers in patients (42). Some scholars had proposed that prolactin can bind to specific receptors on the surface of platelets to enhance platelet activity (43, 44). And prolactin can induce inflammatory response in the body, lead to the activation of a variety of inflammatory mediators and inflammatory cells, and affect endothelial function, thus directly or indirectly promote the formation of thrombus (45, 46). Therefore, we plotted the ROC curve combined with these three indicators for the first time. The results indicate that the combination of DDI, homocysteine and prolactin has a good predictive performance for the incidence of VTE in patients with mental illness.

Referring to the literature, this is the first clinical study to explore the relationship between hyperhomocysteinemia and VTE in patients with mental illness. as mentioned above, previous studies focused on the relationship between hyperhomocysteinemia and VTE in the general population, and the conclusions were different, which may be related to the selection bias of the included population, the different definition of hyperhomocysteinemia and the influence of confounding factors. Secondly, the blood samples in this study were collected before the VTE event, so it can further substantiate the causal relationship between hyperhomocysteinemia and VTE in patients with mental illness. This paper also performed a subgroup analysis according to the thrombus site, and further analyzed the interaction between sex and homocysteine, and then conducted stratified analysis by sex. Based on the study of this paper, we discovered that female mental patients with hyperhomocysteinemia were at high risk of VTE. This study is beneficial for clinicians to detect high-risk patients early and initiate thrombus prevention measures for high-risk patients as soon as possible, so as to effectively reduce poor prognosis.

However, this study also had some limitations, in the subgroup analysis according to the thrombus site, the number of cases in some subgroups was small, especially in the DVT+PE group, so the conclusion may be biased, resulting in underestimating the association between hyperhomocysteinemia and DVT+PE group. Secondly, most of the patients included in this study were over 50 years old, so the conclusions may not be applicable to young patients with mental illness. Additionally, this investigation was a single-center study involving Chinese patients with mental disorders, predominantly those suffering from depression, and owing to the lack of severity assessment in the initial case data, a classification of disease severity was not incorporated into the statistical model. Moreover, in the process of data collection, it was observed that antipsychotic medications varied significantly in their varieties and dosages, so they couldn’t be included in the statistical model. As a result, a binary classification approach was used without dosage information. Due to considerable variability in patient heterogeneity concerning the temporal relationship between fasting blood samples and thrombotic events, this section was not further analyzed in this paper. Finally, the predictors of this paper can not quantitatively evaluate the thrombus risk of patients with mental illness, and larger samples and further studies are needed in the future to develop a unique predictor of thrombus risk for patients with mental illness.

Patients with mental illness are at risk of VTE. Because of their diseases and the influence of taking antipsychotic drugs, they will increase the risk of metabolic syndrome. Therefore, the risk factors of VTE in patients with mental illness may be different from the general population. More studies are needed to explore the risk factors of VTE in patients with mental illness in the future. Further research will help to develop a unique thrombus risk assessment form for patients with mental illness in the future, so that high-risk patients can be identified early and accurate risk stratification can be carried out for patients. For high-risk patients, we should be vigilant, start physical prevention as early as possible (encourage more drinking water, more activities and the use of double lower extremity DVT pumps, etc.), and use drugs when necessary, so as to reduce the occurrence of VTE and improve the clinical prognosis of patients.

## Conclusion

In patients with psychiatric disorders, a strong correlation was found between hyperhomocysteinemia and Pulmonary Embolism (PE). An interaction between homocysteine levels and gender was observed. Hyperhomocysteinemia significantly increases the risk of Venous Thromboembolism (VTE) in female psychiatric patients. Therefore, the conclusion of this article is that female psychiatric patients with hyperhomocysteinemia constitute a high-risk group for VTE. This discovery has important clinical implications, indicating that early preventive measures can be taken for this high-risk group. This will reduce the risk of thromboembolic events during hospitalization for patients with psychiatric disorders, thereby improving their clinical outcomes.

## Data availability statement

The original contributions presented in the study are included in the article/supplementary material. Further inquiries can be directed to the corresponding author.

## Ethics statement

The study passed ethical review by the Ethics Committee of Sir Run Run Shaw Hospital, College of Medicine, Zhejiang University (Study Approval No.0433). The ethics committee waived the requirement of written informed consent for participation due to the retrospective design. The studies were conducted in accordance with the local legislation and institutional requirements.

## Author contributions

JW: Data curation, Investigation, Methodology, Software, Writing – original draft. YZ: Data curation, Investigation, Methodology, Writing – review & editing. KR: Data curation, Investigation, Writing – review & editing. YL: Data curation, Methodology, Writing – review & editing. KY: Supervision, Writing – review & editing.
